# Hatchery supplementation provides a demographic boost but alters age composition of sockeye salmon in Auke Lake, Southeast Alaska

**DOI:** 10.1111/eva.13640

**Published:** 2024-02-07

**Authors:** Megan V. McPhee, Patrick D. Barry, Chris Habicht, Scott C. Vulstek, Joshua R. Russell, William W. Smoker, John E. Joyce, Anthony J. Gharrett

**Affiliations:** ^1^ College of Fisheries and Ocean Sciences University of Alaska Fairbanks Juneau Alaska USA; ^2^ Alaska Fisheries Science Center National Marine Fisheries Service, National Oceanic and Atmospheric Administration Juneau Alaska USA; ^3^ Gene Conservation Lab Alaska Department of Fish & Game Anchorage Alaska USA

**Keywords:** age at maturity, aquaculture, fitness, parentage, reproductive success

## Abstract

Evaluating salmon hatchery supplementation programs requires assessing not only program objectives but identifying potential risks to wild populations as well. Such evaluations can be hampered by difficulty in distinguishing between hatchery‐ and wild‐born returning adults. Here, we conducted 3 years (2011–2013) of experimental hatchery supplementation of sockeye salmon in Auke Lake, Juneau, Alaska where a permanent weir allows sampling and genotyping of every returning adult (2008–2019). We identified both hatchery‐ and wild‐born returning adults with parentage assignment, quantified the productivity (adult offspring/spawner) of hatchery spawners relative to that of wild spawners, and compared run timing, age, and size at age between hatchery‐ and wild‐born adults. Hatchery‐spawning females produced from approximately six to 50 times more returning adults than did naturally spawning females. Supplementation had no discernable effect on run timing and limited consequences for size at age, but we observed a distinct shift to younger age at maturity in the hatchery‐born individuals in all three brood years. The shift appeared to be driven by hatchery‐born fish being more likely to emigrate after one, rather than two, years in the lake but the cause is unknown. In cases when spawning or incubation habitat is limiting sockeye salmon production, hatchery supplementation can be effective for enhancing the number of returning adult fish but not without the risk of phenotypic change in the recipient population, which can be an undesired outcome of hatchery supplementation. This study adds to a growing body of evidence suggesting that phenotypic change within a single generation of captive spawning might be widespread in salmon hatchery programs.

## 1 INTRODUCTION

Hatcheries have become an integral part of salmon management around the Pacific Rim. Salmon hatcheries have objectives and protocols that vary by region and have changed over time. In the Pacific Northwest region of the United States, hatcheries were originally intended to mitigate for freshwater habitat destruction by replacing wild runs with hatchery‐produced fish (Lichatowich, 1999), but currently they may focus more on supplementing declining natural populations and warding off extinction of Evolutionarily Significant Units under the United States Endangered Species Act (e.g., Snake River sockeye salmon *Oncorhynchus nerka*, Johnson et al., 2020). In Japan, chum salmon *O. keta* hatcheries provide commercial fishing opportunities that cannot be supported by wild production due to loss of freshwater and estuarine habitat (Miyakoshi et al., 2012). In Alaska, Canada, and the Russian Far East, where wild salmon runs remain abundant, segregated hatcheries are intended to enhance harvest of salmon and to compensate for years of low natural salmon abundance (Kaev, 2012; Smoker et al., 2000). Hatcheries require substantial resources to operate and pose risks to wild salmon populations (Laikre et al., 2010; Naish et al., 2008); as such the cost–benefit ratio of hatcheries is a fervently debated topic in Pacific salmon management and conservation.

Hatchery enhancement of sockeye salmon in transboundary drainages, which originate in Canada and flow through Southeast Alaska, is mandated by the Pacific Salmon Treaty (PST). Enhancement is conducted jointly by the USA and Canada under an integrated supplementation model (Cuenco, 1994) whereby broodstock is taken from the natural adult population and spawned on site; fertilized eggs are transported to Snettisham Hatchery south of Juneau, Alaska for incubation and thermal otolith marking (Volk et al., 1999). In the following spring, fry weighing approximately 0.15–0.24 g are released into natal rearing lakes to feed, grow, and emigrate volitionally. According to annual reports of the Pacific Salmon Commission Transboundary Technical Committee,^1^ on average 10.1% of the escapement to Tahltan Lake (in the Stikine River drainage) and 1.3% of the Taku River escapement were taken as broodstock from 2016 through 2020. Otolith marks are used to evaluate the contribution of hatchery‐origin fish to harvest and escapement in these transboundary systems.

Further research is necessary to assess the effectiveness of transboundary sockeye salmon supplementation programs. Mathias (2000) and Hyatt et al. (2005) estimated survival for hatchery and wild fry over multiple years in Tatsemenie Lake (in the Taku River drainage) and Tahltan Lake. They found that fry survival through their first summer was consistently lower in hatchery than wild fish in Tatsemenie Lake but consistently higher in hatchery fish in Tahltan Lake, and a similar pattern was seen for survival to smolting. They attributed the differences in relative survival of hatchery fish to ecological differences in the lakes, specifically whether populations were limited by lake rearing habitat or spawning and/or incubation habitat. In Tatsemenie Lake where the sockeye salmon population appears to be limited by lake rearing habitat (possibly in the littoral rather than limnetic zone; Riffe & Mercer, 2006), hatchery supplementation has not been as effective, and the removal of adults from the wild population for hatchery broodstock could be counterproductive if not properly implemented. In Tahltan Lake, hatchery supplementation appears to overcome spawning habitat limitation and effectively provides a demographic boost to the population, at least to the smolt stage. Efficacy in terms of the ultimate goal, increased numbers of returning adults, is less well understood.

The ability to comprehensively evaluate a supplementation program is constrained by methods to distinguish between hatchery‐ and wild‐born individual fish in the field. In transboundary supplementation programs, lethal sampling is required to identify presence (hatchery origin) or absence (wild origin) of a thermal otolith mark. This limits the number of fish that can be sampled, particularly as adult sampling is most efficient at counting weirs when individuals have yet to fully mature and spawn. Otoliths can be obtained from senescent fish (e.g., spawned‐out carcasses), but such sampling is resource‐intensive, does not sample the entire population, and can be size‐ and/or age‐biased (Zhou, 2002). Parentage assignment (Steele et al., 2019) overcomes tagging constraints by using genotypes to assign returning adult salmon to their natal origin. Most studies that use parentage to evaluate hatchery programs focus on the second‐generation (*F*_2_) effects of hatchery supplementation that is, whether the reproductive success of hatchery‐born individuals that spawn in the wild differs from that of wild‐born fish (Koch & Narum, 2021). However, this method is also ideal for evaluating the effectiveness of supplementation in the first (*F*_1_) generation, because productivity can be directly quantified in terms of the number of adults produced per captive‐spawned fish versus that of wild spawners. Furthermore, it allows assessment of the effect of hatchery rearing on adult phenotypes, such as run timing or body size, which is an important but understudied aspect of hatchery program evaluations. Here, we used parentage assignment to quantify the relative productivity of hatchery supplementation of sockeye salmon as practiced under the Pacific Salmon Treaty (PST). We conducted experimental captive spawning of sockeye salmon in Auke Lake, Juneau, Alaska, over three successive brood years (2011–2013) following the hatchery supplementation model practiced in transboundary systems pursuant to the PST. We comprehensively sampled parents and returning adult offspring at the weir on Auke Creek (2008–2019) and used genotypes to assign returning adult offspring to individual parents. This allowed us to compare productivity, defined as the number of returning adult offspring per female spawner, between hatchery and natural spawners. We determined whether run timing, age, and size at age differed between hatchery and natural‐origin offspring; such differences could be problematic if a goal of supplementation is to minimize phenotypic and genetic changes to the recipient population. Near‐census genotyping of Auke Lake sockeye salmon allowed us to assess the demographic effects of hatchery supplementation and represents a first step toward examining next‐generation effects of hatchery supplementation (i.e., reproductive success of *F*_1_ spawners) in the future.

## 2 METHODS

### 2.1 Adult sampling and hatchery supplementation

**FIGURE 1:**
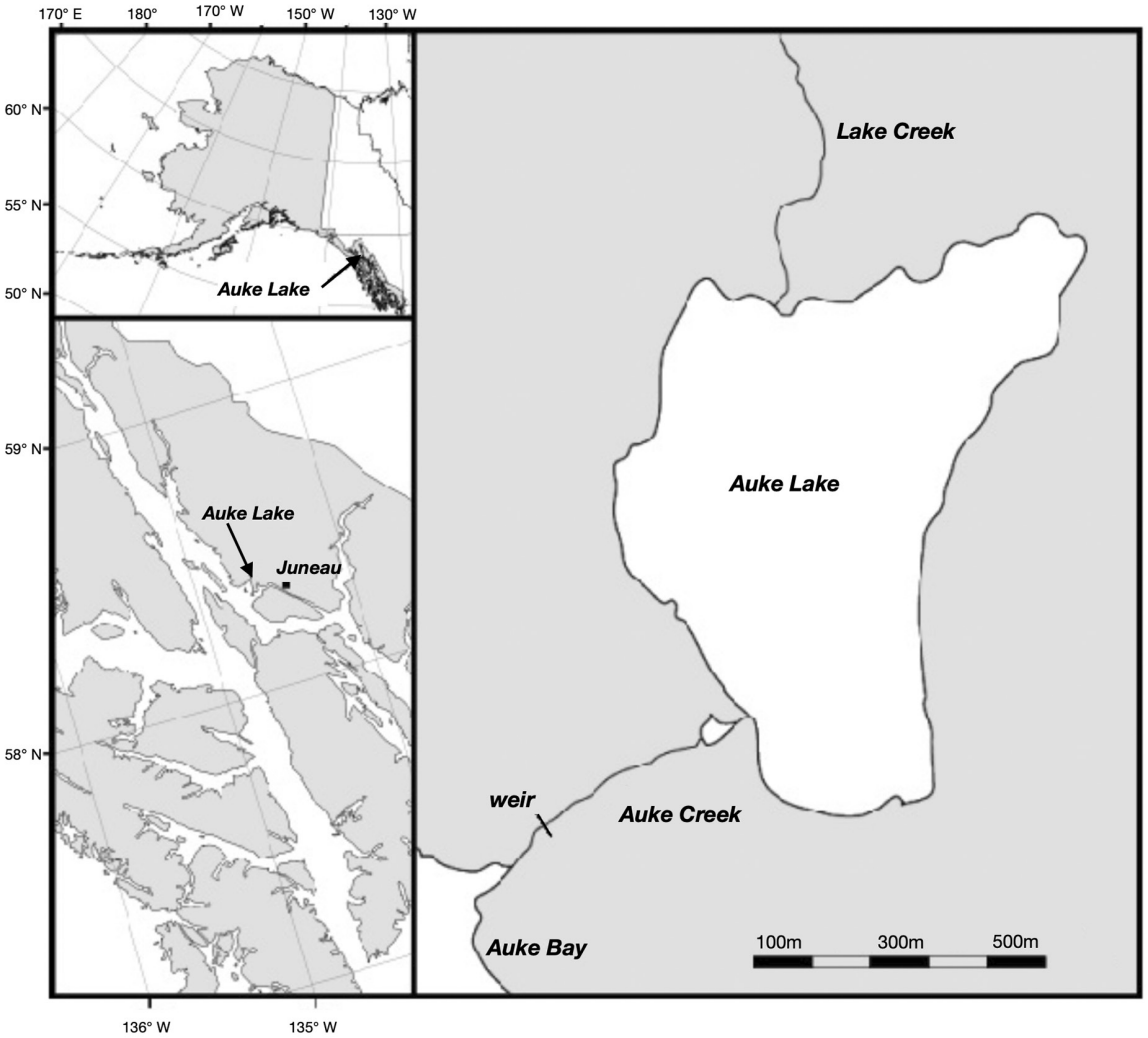
Map of Auke Lake near Juneau, Alaska, showing the location of the weir, where returning adult sockeye salmon were sampled, and Lake Creek, where the majority of fish spawn.

This study took place in the Auke Lake drainage in Juneau, Alaska (Figure 1). Auke Lake is small (≈67 ha) and located 323 m from the coast (Manhard et al., 2018). A permanent weir, located at 58.381^o^N, 134.641^o^W and operated by the National Ocean and Atmospheric Administration (NOAA), spans the outlet of Auke Creek and is configured to sample downstream juvenile migrants in the spring and returning adult salmon in the summer and fall. Juvenile and adult sockeye salmon, pink salmon *O. gorbuscha*, chum salmon, coho salmon *O. kisutch*, as well as cutthroat trout *O. clarkii* and Dolly Varden *Salvelinus malma*, have been counted at the weir annually since 1980. Hatchery supplementation of sockeye salmon was previously conducted in Auke Lake with age‐0 smolts released 1988–1992 (Heard et al., 2013). Following cessation of that program, sockeye salmon returning to Auke Lake originated entirely from natural spawning except for the three brood years (2011–2013) of experimental supplementation conducted for this study.

Each returning adult sockeye salmon was captured at the weir, identified based on the appearance of the snout and vent as a female, regular‐sized male, or “jack” (precocious male ≤400 mm mid‐eye to fork length) male, and had an axillary process removed and stored in 95% ethanol for subsequent DNA extraction and genotyping. A subsample of individuals was also sampled for mid‐eye to fork length (mm) and had a scale removed for age determination (J. Russell, NOAA, Unpublished data).

**FIGURE 2:**
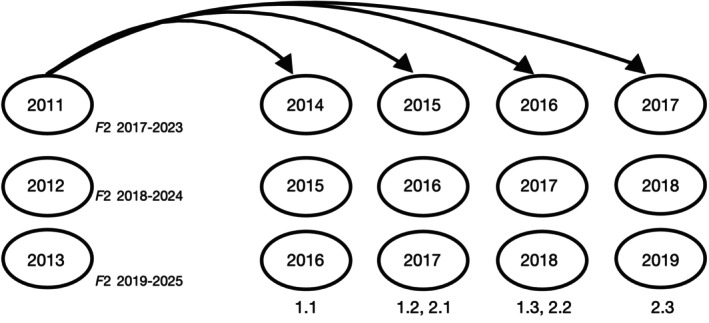
Schematic showing the return years of adult offspring resulting from brood years 2011–2013, when supplementation was conducted. Ages in European age notation are shown below the offspring bubbles (e.g., “1.2” indicates one scale annulus in freshwater and two annuli in the ocean prior to maturation at age 4).

Over three successive years (2011–2013), sockeye salmon were artificially spawned and their progeny were reared to the early fry stage prior to release into Auke Lake. A small number of adults (<50 per year, averaging 2.5% of the total run annually; Table 1) were captured at the weir, retained for broodstock, held at the hatchery in tanks (≈6 weeks) until mature, and subsequently spawned. The target for broodstock was 30 females and 15 males with a 2:1 breeding design (each male crossed with two females). Fertilized eggs were placed in vertical flow incubators fed by water from a 2.3 m deep intake in Auke Lake. Emergent fry were ponded and given starter feed (Pacific Bio‐Products, Inc.) at the manufacturer's size‐ and temperature‐dependent recommendations for optimal growth; this captive rearing period lasted for ≈4 weeks. Each year, ≈50,000 fry (≈0.3 g) were released into several locations in the limnetic zone of Auke Lake, where they were allowed to feed and grow alongside wild‐born individuals and emigrate volitionally to the ocean after 1 or 2 years in freshwater. Sockeye salmon return to Auke Creek 3–6 years after birth (Kovach et al., 2014), so returning adults that were born in captivity would return over the years 2014–2019 (Figure 2).

**TABLE 1:** Numbers of adult sockeye salmon (Nf, females; Nm, regular‐sized males; Nj, “jacks”/precocious males, and total) returning each year, as counted at the Auke Creek weir, as well as the number (and percentage) of adults sampled for scales.

Return year	Nf	Nm	Nj	Total adults	Scale samples	Parent years	Nmax
2011	1303	998	122	2423	320 (13.2%)	—	—
2012	748	486	49	1556	201 (12.9%)	—	—
2013	1043	910	107	2060	188 (9.1%)	—	—
2014	1762	1583	98	3443	402 (11.7%)	2008–2011	5150
2015	2507	2082	131	4720	360 (7.6%)	2009–2012	5305
2016	1281	1220	18	2519	307 (12.2%)	2010–2013	4255
2017	2011	1612	40	3663	360 (9.8%)	2011–2014	4780
2018	446	465	12	923	271 (29.4%)	2012–2015	6185
2019	615	466	23	1106	240 (21.7%)	2013–2016	6690

*Note*: Also shown are the relevant parent years and the FRANz parameter “Nmax” (twice the estimated number of potential parents for adults returning in a given year).

Realized broodstock numbers varied slightly from the 30 females and 15 males targeted each year due to prespawn mortality or incomplete maturation at the time of attempted spawning. Ultimately, 30 females and 14 males spawned in 2011, 23 females and nine males spawned in 2012, and 25 females and 14 males spawned in 2013. No jacks were included as hatchery broodstock, but otherwise hatchery spawners were similar in size to a measured subsample of wild spawners except in 2012, when the average length of hatchery spawners was 32 mm smaller than that of wild spawners (*t*_42_ = −4.3, *p* = 0.0001; Figure S1). The dates of broodstock capture deviated slightly from the average return time of wild spawners. In 2011, hatchery spawners were captured at the weir on 25–26 July, and the median return date of wild spawners was 26 July. In 2012, hatchery spawners were captured on July 17–18, 2012, which was 7–8 days later than the median return date of wild spawners. In 2013, hatchery spawners were captured on July 10–11, 2013, 2–3 days earlier than the median return date of wild spawners (Figure S1).

### 2.2 Genotyping

Genotyping was conducted at the Gene Conservation Lab of Alaska Department of Fish and Game. Genomic DNA was extracted using DNeasy® 96 Tissue Kit (Qiagen). Samples were genotyped at nine short tandem repeat (STR) and 45 single nucleotide polymorphism (SNP) markers (Table S1). Taqman assays and the Fluidigm Biomark 96.96 and 48.48 Dynamic Arrays were used to genotype the SNPs, following the methods outlined in Seeb et al. (2009). Amplification of STR loci was carried out in 10 μL reaction volumes and PCR fragments were identified on an Applied Biosystems 3730 capillary DNA sequencer. PCR bands were visualized and separated into bin sets using AB GeneMapper software v4.0 and automated binning was subsequently confirmed or corrected manually. A subsample (eight of every 95 individuals) was re‐extracted and genotyped to quantify genotyping error rate.

We checked the genotype data for deviations from Hardy–Weinberg proportions, which could indicate null alleles if *F*_IS_ values were consistently positive in all sample years, and for linkage disequilibrium, which could indicate physical linkage if detected in all sample years. Deviations from Hardy–Weinberg expected proportions within each sample year were analyzed with *χ*‐square tests in the R package pegas (Paradis, 2010). Pseudo‐exact tests were used to test for nonrandom associations of alleles between all possible locus pairs, both within years and over all years combined, in genepop (Rousset, 2008) v. 1.2.2 for R. These analyses were conducted on the individuals that were successfully genotyped at all 54 loci.

### 2.3 Parent–offspring assignment and relative productivity

Parentage analysis was conducted with FRANz (Riester et al., 2009) on an individual return‐year basis, where adult offspring returning in a given year were compared to all potential parents that spawned from 3 to 6 years prior. The average empirical estimate of genotyping error rate was 0.001 for SNPs and 0.0001 for STRs. However, preliminary analysis of assignments from known hatchery crosses (not shown) revealed that using a genotyping error rate of 0.001 produced more false negatives than did a rate of 0.005, so we used 0.005 as the genotyping error rate for both marker types for parentage inference. The minimum allowed number of genotyped SNP and STR loci was 40 (of 54 total) and the maximum number of mismatches allowed was two for a parent–offspring dyad and three for a sire‐dam–offspring triad, based on simulation results of FRANz. For each run, the maximum number of potential parents (the “Nmax” parameter in FRANz) was given as the number of adults observed at the weir divided by two (because in FRANz Nmax = number of males = number of females), summed over all potential brood years. We added 5% to this number to account for uncertainty in weir counts and to reduce the chance of false‐positive assignments (Table 1); this sum was rounded up to the nearest five individuals. The assumption of an equal sex ratio was reasonable for Auke Creek sockeye salmon (Table 1), and preliminary analyses found that increasing Nmax to 8% or 10% of the actual weir counts produced the same parentage assignments as the 5% increase (data not shown).

We used CKMRsim v. 0.1.1.999 (github.com/eriqande/CKMRsim/; Baetscher et al., 2018) to evaluate the power of our suite of markers to adequately assign parentage and to identify an optimal log‐likelihood ratio (LOD) threshold for accepting assignments. We based our simulations on allele frequencies estimated from all samples 2008–2019 and a genotyping error rate of 0.005. Results of the simulations showed clear separation of the LOD values between parent–offspring and unrelated pairs (Figure S2). Because we had nearly complete population sampling, we wanted to avoid both false positive and false negative assignments when calculating reproductive success. A log‐likelihood ratio threshold of ≥4.5 optimized the trade‐off between false negative and false positive rates (0.02 and 2.3 × 10^−4^, respectively); accordingly, we used this threshold for accepting parent–offspring (dyad) assignments when calculating relative reproductive success. For analysis of phenotypic differences between hatchery and wild offspring, we wished to further reduce false positive rates and therefore based those analyses on dyad assignments with LOD ≥9 (false positive rate = 1 × 10^−5^). We also evaluated productivity when assignments were accepted with no LOD cutoff to evaluate potential bias introduced by such thresholds (Ford & Williamson, 2010).

Field identification of the sex of sockeye salmon in Auke Creek was not free from error, particularly early in the season when secondary sexual characteristics are less developed. In some cases, we ended up with triad assignments consisting of two “female” or two “male” parents based on field identifications. Where possible, we examined other mates to infer the most parsimonious sex of the focal individuals. In monogamous pairs, sex was assigned randomly. In cases where sex could not be ascribed parsimoniously (i.e., the individuals appeared to be truly the same sex based on inference from other mates), we rejected the parent assignment with the lower LOD.

Adults could be assigned zero offspring either because they produced no surviving offspring, they had insufficient genotype information to be assigned offspring, or their offspring had insufficient genotype information to be assigned to parents. We assumed that offspring with insufficient genotype information were distributed proportionately between adults with and without sufficient genotype information and were thus ignored in calculations of productivity. Some individuals were missing genotypes at all STR loci but were successfully genotyped for the majority of SNPs. Results of simulations in CKMRsim, as described above, indicated that the STRs were necessary for accurate parentage assignment (Figure S2). Overall, potential parents missing genotypes at ≥5 STRs or ≥ 22 SNPs were excluded from productivity calculations, as were any individuals with fewer than 40 genotyped loci in total.

There were several instances where hatchery spawners lacked genotypic information, so their offspring would be assigned only to their mate. Although hatchery records would allow assignment of offspring to those parents with missing genotypes, we chose not to assign offspring to these individuals to avoid bias in relative productivity calculations that could arise from treating hatchery and wild parentage assignments differently. Overall, successful genotyping rates varied across brood years but not between hatchery and wild adults, suggesting that bias was minimal (Table S2).

Productivity was calculated as the average number of returning adult offspring assigned to hatchery and wild parents, for each brood year and sex separately. We focused the presentation and interpretation of our results on the productivity of females, though, because the two females:one male cross design artificially inflated the difference in productivity between sexes in the hatchery spawners. Relative productivity (RP) was calculated as the productivity of hatchery females divided by that of wild females, and 95% confidence intervals were calculated as described in Kalinowski and Taper (2005) using an R function modified from Shedd et al. (2022).

### 2.4 Phenotypic differences between hatchery and wild offspring

We compared run timing, age at maturity, and size at age between hatchery‐ and wild‐born adult offspring. We limited the analysis of offspring phenotypes by origin to those assigned to at least one parent in brood years 2011–2013 with LOD ≥9, which was a more stringent threshold for positive assignment than the threshold used for RP calculations. We first compared the phenotypes of hatchery‐born individuals to all wild‐born individuals originating from the same brood year. Next, we compared the phenotypes of hatchery‐born individuals to only the wild‐born offspring that had at least one parent that returned during the same 2‐day period over which hatchery parents were collected. This allowed us to investigate whether any differences in phenotypes of hatchery‐ and wild‐born salmon were related to return timing of their parents, since adult traits such as reproductive life span or body size often vary over the course of a run (Doctor & Quinn, 2009; McPhee & Quinn, 1998). These analyses were conducted for each brood year separately.

Run timing was recorded as the day of year an individual was captured at the weir. Run timing differences between hatchery‐ and wild‐born offspring were analyzed by return year, since run timing in sockeye salmon is influenced by environmental conditions during the year of return (Hodgson et al., 2006). We used two‐sided Kolmogorov–Smirnov tests to determine whether run timing distributions differed significantly between origin and applied the Holm–Bonferroni correction (Holm, 1979) to adjust for the family‐wise error rate.

Age at maturity was inferred primarily from parent assignment. Juvenile sockeye salmon rear in Auke Lake either 1 or 2 years prior to emigration (Kovach et al., 2014), and because only a subset of individuals was sampled for scales (Table 1), we could not infer freshwater and ocean ages for most of the returning adults. The age composition of adult offspring was compared between origins within brood years using χ‐square tests and applying the Holm–Bonferroni correction. Although there were four age classes of adults returning to Auke Creek (3–6 years old), age‐3 offspring were rare, so ages 3 and 4 were pooled for the χ‐square tests. We secondarily explored variation in freshwater and saltwater age by origin using a subsample of individuals for which scale ages were available (J. Russell, NOAA, Unpublished data) and agreed with parentage‐based ages. We compared the proportion of individuals that emigrated after 1 year in fresh water, relative to those that emigrated after 2 years, between hatchery‐ and wild‐born individuals. Fisher's exact tests were performed for each brood year and for all brood years combined and the Holm–Bonferroni correction was applied.

Comparisons of size at age were based on a sample of individuals for which mid‐eye to fork length (MEFL; mm) was measured and scale ages agreed with total ages inferred from parentage assignment. Analysis was further limited to the scale ages found in both hatchery‐ and wild‐born offspring, that is, 1.2, 1.3, 2.2, and 2.3, where the integer before the decimal represents number of years in freshwater and the integer after represents the number of years in saltwater prior to maturation (Koo, 1962). We tested for differences in size at age between hatchery‐ and wild‐born individuals by fitting linear models including origin (*O*, hatchery vs. wild), sex (*S*), and scale age (*SA*; as a ranked factor) as explanatory variables:

$$\text{MEFL}=\alpha +\beta_{O}+\beta_{S}+\beta_{\mathrm{SA}}+\varepsilon .$$

Preliminary analyses detected significant interactions between brood year and each of the other explanatory variables, so to facilitate interpretation, we modeled each brood year separately. Model assumptions were validated by visual inspection of the residuals for normality and lack of heteroscedasticity (Zurr et al., 2009). Small sample sizes prevented us from repeating this analysis using only offspring of wild parents that passed the weir on the same date as hatchery parents.

## 3 RESULTS

### 3.1 Genotyping and parent–offspring assignment

Over return years 2008–2019, we attempted to genotype 26,776 individuals and obtained sufficient genotypic data (≥40 loci) for 25,867 (99.6%) of these. We found deviations from Hardy–Weinberg expected genotype proportions: of the 594 χ‐square tests performed, 116 (19.5%) had *p* < 0.05. Seven STRs and 19 SNPs had tests with *p* < 0.01. Of these, two STR loci (*Oki10* and *Ssa419*) showed consistently positive *F*_IS_ values across sample years (ranging from 0.005 to 0.036 for *Oki10* and 0.013 to 0.040 for *Ssa419*), suggesting the likely presence of null alleles in these two loci. Testing for linkage disequilibrium between all locus pairs in all sample years combined involved 1431 exact tests, of which 64% had *p* < 0.05. Of the 15,741 tests of all locus pairs within each sample year, 25% had *p* < 0.05. However, only one locus pair (STR *One8* and SNP *One_U1012_68*) showed strong evidence of being physically linked, with highly significant tests (*p* < 0.0001) for departure from linkage equilibrium in each sample year. Given the low information value of individuals SNPs relative to STRs, we chose to retain both loci in parentage analyses.

We compared 13,102 adult offspring that returned over 6 years (2014–2019) to 20,349 potential parents over nine brood years (2008–2016). In total, 12,874 offspring (98.3%) were assigned to at least one parent with LOD ≥4.5, of which 6800 offspring (51.9%) were assigned to at least one parent from brood years 2011–2013. Increasing the threshold for accepting dyad assignments to LOD ≥9 resulted in a small reduction in assignments, with 6441 offspring (49.2%) assigned to at least one parent from brood years 2011–2013. Crosses that initially appeared to be same‐sex pairs comprised 6.7%, 4.1%, and 7.2% of the assignments made with the LOD ≥4.5 threshold in brood years 2011, 2012, and 2013, respectively.

### 3.2 Reproductive success

**FIGURE 3:**
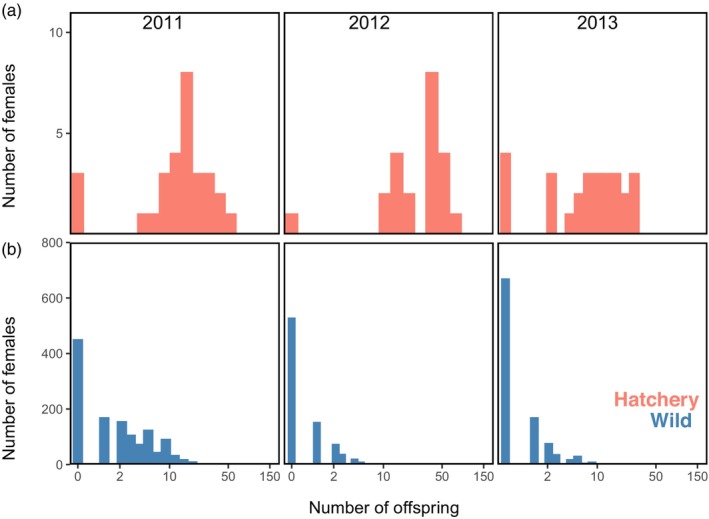
Distribution of the number of returning adult offspring per female by parental type (hatchery in pink; wild in blue) and brood year. The top panel (a) includes parents that produced no offspring and the bottom panel (b) only includes females that produced at least one offspring. Note the varying *y* scale between hatchery and wild categories and the natural‐log scaling of the *x* axis.

Hatchery supplementation produced far more returning adult offspring per spawner than did wild spawning (Figure 3). In all three brood years, hatchery females produced many more returning adult offspring than did wild females, translating into RP values >>1, with no 95% CIs including unity (Table 2). The average number of adult offspring per female was below replacement for wild adults in 2012 and 2013, with >60% of wild spawners producing no returning adult offspring in those 2 years. Relative productivity was particularly high in 2012 when hatchery females produced ≈50 times more offspring on average than wild females. The maximum individual reproductive success was 71 offspring from a hatchery female, 24 offspring from a wild female, 131 offspring by a hatchery male, and 25 offspring by a wild male, although some individuals of both sexes and spawning types produced zero offspring. The hatchery design of two females per male resulted in the productivity of hatchery males being approximately twice that of hatchery females (not shown), so we focused primarily on the relative productivity of female spawners.

**TABLE 2:** Number of female spawners (dams) and total number of adult offspring by parental type and relative productivity (with 95% confidence intervals in parentheses) by brood year.

Brood year	Hatchery		Wild		Relative productivity
	Dams	Offspring	Dams	Offspring	
2011	29	504	1263	3653	6.0 (5.5–6.6)
2012	22	701	815	534	48.6 (43.5–54.4)
2013	27	286	1006	770	13.8 (12.1–15.8)

The distributions of dyad LOD scores for all assignments (LOD > 0) were similar between hatchery and wild parents over all three brood years except for the tails of the distributions (Figure S3). Wild parent assignments had more extreme positive and negative LOD values, but the averages were similar and not always greater for hatchery parent assignments. Estimates of relative productivity between hatchery and wild fish differed little between assignments made with no LOD threshold versus a threshold of LOD ≥4.5 (Table S3), suggesting that our use of a threshold for accepting assignments produced minimal bias in RP.

### 3.3 Phenotypic differences in offspring

**FIGURE 4:**
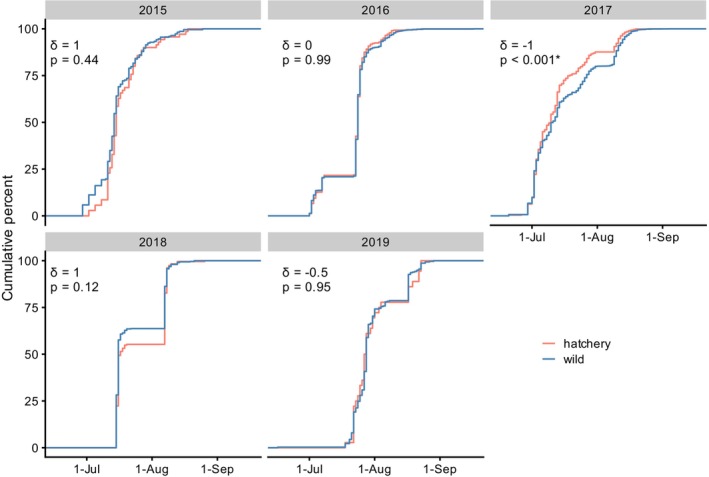
Cumulative proportion of the adult salmon run by date for each return year and origin (hatchery or wild). Also shown are *δ* (the difference between the median dates of hatchery vs. wild origin offspring, where a negative value represents earlier median date of hatchery‐origin return) and *p* (the probability of a difference between median date of return [two‐sided Kolmogorov–Smirnov test]).

Hatchery rearing had no apparent effect on run timing. Of all return years 2015–2019, the distribution of return dates differed significantly only in 2017 (D = 0.107, and *p* = 2.6 × 10^−6^), and in that case, the median return date of hatchery‐born fish was 1 day earlier than that of wild‐born fish (Figure 4). When comparisons were limited to those with wild parents sampled on the same dates as hatchery parents, the results were similar: run timing of wild‐ and hatchery‐born offspring differed significantly only in return year 2017 and the median return date of hatchery‐born fish was 2 days earlier than that of wild‐born individuals (Figure S4).

Hatchery‐born offspring tended to be younger than wild‐born offspring: age 5 offspring dominated the return from hatchery parents, whereas the wild‐born offspring were more evenly distributed between ages 5 and 6. Differences in age composition as determined by parentage were significant in all three brood years, regardless of whether we compared offspring from all wild parents or just those from wild parents that returned on the same dates as the hatchery parents (Table 3). In the scale‐aged individuals with concordant parentage‐ and scale‐based ages (265, 98, and 120 individuals from brood years 2011, 2012, and 2013, comprising 10.9%, 6.3% and 5.8% of the adult returns, respectively), we found that freshwater age 1 was more frequent than freshwater age 2 in individuals of hatchery origin, while the opposite was true for wild‐born fish. The difference in freshwater age between hatchery‐ and wild‐born fish was statistically significant in all brood years, regardless of whether we analyzed wild‐born offspring from all parents or only the parents that returned during the days that hatchery broodstock were collected (Table 4).

**TABLE 3:** Proportion of adult offspring within each age class by brood year and origin (hatchery vs. wild) and results of chi‐square test for homogeneity of proportions between origins (hatchery or wild; two degrees of freedom for all comparisons).

	Age 3	Age 4	Age 5	Age 6	*N*	*χ* ^2^	*p*
Wild (all)							
2011	0.013	0.060	0.327	0.600	3642	183.14	<0.0001
2012	0.009	0.040	0.653	0.297	528	125.95	<0.0001
2013	0.010	0.070	0.487	0.433	725	92.74	<0.0001
Wild (broodstock dates)							
2011	0.011	0.064	0.320	0.605	2015	169.2	<0.0001
2012	0.029	0.082	0.582	0.306	170	72.9	<0.0001
2013	0.021	0.075	0.464	0.439	239	64.5	<0.0001
Hatchery							
2011	0	0.125	0.581	0.294	554		
2012	0.001	0.145	0.782	0.072	711		
2013	0	0.204	0.667	0.129	279		

*Note*: Age 3 and age 4 offspring were pooled for significance testing. “Wild (broodstock dates)” refers to the wild parents that were sampled at the weir over the same 2 days in each brood year that hatchery parents were collected.

**TABLE 4:** Proportion of adult offspring that spent one (FW‐1) or two (FW‐2) years in freshwater prior to going to sea as determined from scale samples, by parental type (wild and hatchery) and brood year.

Parental type	Brood year	FW‐1	FW‐2	*N*	*p*
Wild (all)	2011	0.256	0.744	234	<0.0001
	2012	0.245	0.755	53	<0.0001
	2013	0.228	0.772	92	0.009
Wild (broodstock dates)	2011	0.238	0.762	126	<0.0001
	2012	0.118	0.882	17	0.0001
	2013	0.351	0.649	37	0.31
Hatchery	2011	0.871	0.129	31	
	2012	0.689	0.311	45	
	2013	0.500	0.500	28	

*Note*: Also shown are sample size (*N*) and *p*‐values from Fisher's exact tests for differences in proportions between the nominal wild sample and hatchery sample with one degree of freedom.

**FIGURE 5:**
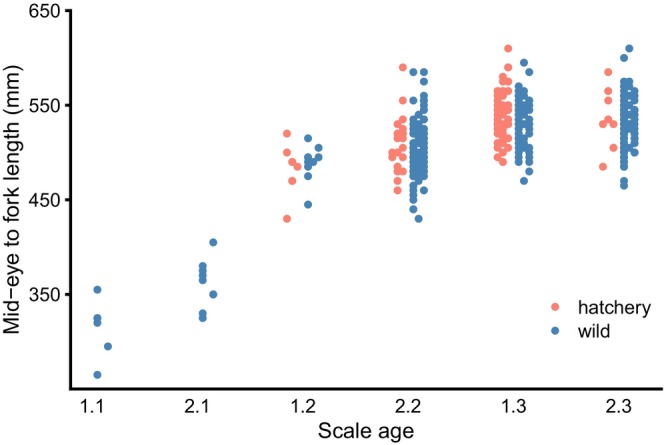
Distribution of size by age (based on scales) between hatchery and wild origin adult offspring. Within an age class and origin, points are arranged horizontally by brood year (left to right, 2011–2013). No offspring from brood year 2012 with scale ages 1.1 or 2.1 were sampled.

Statistical analysis of size at age was limited to older fish (scale ages 1.2, 2.2, 1.3, and 2.3) because we detected no 1.1 or 2.1 hatchery offspring in the sample of scale‐aged fish. Size at age did not differ significantly between hatchery‐ and wild‐born fish resulting from brood years 2011 and 2012, but hatchery‐born individuals tended to be larger (*p* = 0.007) than wild‐born individuals in brood year 2013 after accounting for sex and scale age (Figure 5). Complete size‐at‐age modeling results are given in Table S4.

## 4 DISCUSSION

When evaluating salmon supplementation programs, it is important to quantify whether hatchery spawners produce more returning adults on average than do wild spawners. If the relative productivity of hatchery to wild spawners is <1, then supplementation goals are not being met and the program may be harming the wild population through broodstock removal (Hyatt et al., 2005; Mathias, 2000). Here, we evaluated the relative productivity of sockeye salmon supplementation in Auke Lake over 3 years, finding that hatchery spawning was highly effective with relative productivity (adult offspring per female) ranging from 6.0 in 2011 to 48.6 in 2012.

It is also important to evaluate the phenotypic effects of salmon hatchery programs. We observed no differences in return timing and saw hatchery‐wild differences in size at age in only one brood year (2013). However, hatchery‐born fish returned at a younger age on average than their wild counterparts. This phenotypic shift within a single generation is problematic if a goal of supplementation is to minimize changes in the recipient natural population. These results add to the increasing awareness that hatchery supplementation can change phenotypes within a single generation (e.g., Horreo et al., 2018), and as such should be implemented with caution and include monitoring of phenotypes in subsequent generations.

### 4.1 Relative productivity

Much like the Tahltan Lake supplementation program, the demographic success of sockeye salmon supplementation in Auke Lake was likely due to spawning and/or incubation habitat limiting sockeye salmon production in this system. The vast majority of wild sockeye salmon spawn in one inlet creek (Lake Creek) with typically <10% of individuals spawning in additional inlet creeks and on the lakeshore (Bucaria, 1968; Fukushima et al., 1997; Ray et al., 2015). Low flows can periodically block access to Lake Creek (P. Barry, NOAA (Personal communication, June 18, 2021)) and there are natural barriers to upstream fish passage ≈2 km above the creek's mouth,^2^ so the amount of available spawning habitat appears to be quite small, approximately 4 km of stream. Our findings are consistent with an earlier hatchery experiment conducted with Auke Lake sockeye salmon (1988–1992), when age‐0 hatchery fry were released directly into saltwater or following a short period of saltwater netpen rearing (Heard et al., 2013). In that study, hatchery spawners contributed substantially to the adult return, indicating that the considerable demographic benefits of incubation in captivity extended into adulthood despite the lack of a natural lake‐rearing period and release at small size. Density dependence for the adult‐to‐smolt phase was demonstrated in Auke Creek sockeye salmon prior to recent supplementation (Fukushima et al., 1997; Kovach et al., 2014); our results suggest the density dependence is primarily during incubation rather than during lake rearing.

Additional insight into the reasons for high relative productivity can be gained by examining variation in reproductive success across the three brood years of our study. Wild productivity exceeded replacement only in 2011. In brood year 2012, the year with the highest RP, 31 hatchery spawners produced more returning adult offspring in total than did ≈1500 wild spawners. Interestingly, this was the year in which hatchery spawners were significantly smaller than wild spawners and were collected later than the peak run timing of the wild spawners, indicating that the average values for size and timing were not the fittest phenotypes in 2012. That year, 80 adult sockeye salmon were radio‐tagged and tracked as part of a separate study (Ray et al., 2015), and those authors noted that five out of six reads that they monitored on the delta of Lake Creek appeared to be scoured by an autumn flood event. Environmental data for Lake Creek were not available, but Auke Creek, the lake's outlet, is monitored daily for water temperature and gage height, which are broadly indicative of conditions in Lake Creek^2^. Temperature and gage height data during the return migration and spawning (mid‐summer to early fall) and incubation (mid‐fall to early winter) show that 2011 was generally similar or intermediate to conditions in 2012 and 2013, but during the tail end of the return migration Auke Creek was cooler in 2011 (Figure S5). Elevated temperatures are associated with increased en route and prespawning mortality in sockeye salmon (Barnett et al., 2020; Hinch et al., 2012), raising the hypothesis that below‐replacement recruitment in 2012 and 2013 was due to thermal stress during migration and spawning. Hatchery spawners were captured during the middle of the run and held in cool lake water (≈7°C–13°C; NOAA, Unpublished data) prior to spawning, so they might have been protected from thermal stress. Like elsewhere across the range of sockeye salmon, Auke Creek is experiencing a warming trend (Taylor, 2008; Vulstek et al., 2023) and years with poor natural recruitment are likely to become more frequent in the future.

Our results are not directly comparable to most studies that have used parentage to evaluate reproductive success in Pacific salmon, because we focused on the reproductive success of the first generation of hatchery spawners, whereas the majority of adult‐to‐adult reproductive success studies have measured the reproductive success of the second generation, that is, fish born in the hatchery but spawning in the wild (Christie et al., 2014; Koch & Narum, 2021). In terms of evaluating the conservation goals of a supplementation‐style hatchery program, it is important to evaluate the RRS of hatchery‐born fish when they return and spawn in the wild, because it determines the effects of hatchery supplementation on the mean fitness (productivity) of the natural population in subsequent generations (e.g., O'Sullivan et al., 2020). Our study system is well poised to address that question in the future, once all adult progeny of the first generation have returned (the oldest descendants of the 2013 grandparents will return in 2025). Our study demonstrates the additional utility of parentage assignment for evaluating a primary goal of hatchery propagation (i.e., producing additional fish for harvest) by quantifying reproductive success in the first generation. We note that such studies are only feasible when it is possible to sample and genotype a large proportion of the adult population; the permanent weir on Auke Creek made that possible in our case.

### 4.2 Phenotypic consequences of hatchery supplementation

We detected no differences between wild‐ and hatchery‐born fish in run timing even after accounting for the potential influence of hatchery broodstock collection date. Run timing is highly episodic in Auke Creek; during times of low flow and high temperatures, returning fish will mill at the mouth and then ascend in large numbers when rain events cause flows to increase (Barry, 2021). Hatchery spawners were collected on two consecutive days each year; these days corresponded with peak returns of the wild spawners in 2011 and 2013 but followed the peak by about 1 week in 2012 (Figure S1). A preliminary analysis indicated that the narrow‐sense heritability of run timing in Auke Creek sockeye salmon, at least as defined by date of capture at the weir, is ≈0.2 (M. McPhee, UAF, Unpublished data). This value is lower than the median *h* ^2^ estimate for phenological traits in salmonids (0.51; Carlson & Seamons, 2008). Any allelic differences in the timing of return to the mouth of Auke Creek might have been outweighed by the important role of stream flow in triggering stream entry, as well as shared oceanic conditions during the year of homeward migration (Hodgson et al., 2006). Similarly, shared conditions in the ocean where salmon put on more than 99% of their body mass could explain the lack of consistent differences in size at age between hatchery and wild origin fish. It is not clear why hatchery‐born fish tended to be slightly larger than wild‐born fish from brood year 2013, but the lack of an effect in 2011 and 2012 suggests it is not an inherent consequence of captive breeding and early rearing.

In contrast to run timing and size at age, supplementation of Auke Lake sockeye salmon produced a notable shift in the hatchery‐born population toward younger age at maturity within a single generation. This result is concerning, because minimizing phenotypic change is typically a goal of a supplementation‐style hatchery (Osborne et al., 2020). We were unable to determine the relative importance of plasticity and allelic variation for the change in age structure but changes in phenotype, even if plastic, can alter selective regimes causing evolutionary change in subsequent generations (Caspi et al., 2022; Price et al., 2003). Additionally, because the genetically effective population size (*N*_e_) of semelparous salmon is a product of the effective number of breeders (*N*_b_) and generation length (Waples, 1990), reducing the average age at maturity could exacerbate negative effects of supplementation on genetic diversity. A detailed treatment of the effects of supplementation on *N*_b_, *N*_e_, and genetic diversity in the recipient Auke Lake sockeye salmon population was beyond the scope of this article, but it is the subject of future analyses.

The observed change in the age structure of hatchery‐born individuals could result from heritable differences in life‐history schedule (Iwamoto et al., 1984, Hankin et al., 2009, Heath et al., 2002) coupled with unintentional selection for specific traits in the hatchery broodstock (McLean et al., 2005), higher mortality of hatchery‐born individuals from the first year in freshwater to the second (Van Doornik et al., 2021), or from some aspect of hatchery rearing leading to younger age at smoltification (Larsen et al., 2019). To the best of our knowledge, none of these mechanisms have been investigated in sockeye salmon. There was no consistent bias toward smaller adults in the Auke Lake broodstock (Figure S1) except that the youngest males (“jacks”) were not spawned. If jacks have a heritable tendency to spend 2 years rather than one rearing in the lake, excluding jacks could have contributed to the shift in age structure. An effect of hatchery rearing on early growth trajectory is another plausible explanation for why hatchery‐born fish tended to spend less time in freshwater than wild‐born fish. Faster freshwater growth is associated with younger age at smoltification in wild sockeye salmon populations (Koenings & Burkett, 1987; Tillotson & Quinn, 2016). Hatchery embryos were incubated in water drawn relatively deep in the lake, so they might have experienced warmer temperatures in winter and less variability in temperature and dissolved oxygen across the incubation period. More benign conditions in captivity might have supported better yolk conversion efficiency in hatchery embryos, allowing them to grow larger than their wild counterparts (Bams, 1969). Hatchery fry might have been ponded earlier in the year than when wild fry were switching to exogenous feeding, and the initial feeding on a diet formulated for optimal growth might have put them on a faster growth trajectory than wild fish. If such growth advantages persisted through the first summer in the lake, this could translate into a higher probability of emigrating after 1 year than two for hatchery‐born individuals. Without data on the natural incubation and rearing conditions in the Auke Lake drainage, we cannot confirm that hatchery fish grew faster initially, nor can we assume that an initial growth advantage was carried through the growing season (e.g., Hyatt et al., 2005; Sturdevant et al., 2012). We also cannot rule out other effects of hatchery rearing on life history. For example, hatchery rearing is known to induce epigenetic modifications in salmon, although the functional implications and the degree to which these modifications are transmitted across generations are not well characterized (Le Luyer et al., 2017; Leitwein et al., 2021; Rodriguez Barreto et al., 2019).

Our interpretation of phenotypic effects comes with several caveats. First, we only analyzed a handful of phenotypes for which data were available. It is possible that hatchery rearing affected other phenotypes that went unmeasured. As such, care should be taken to monitor a suite of phenotypes related to fitness, such as body size, body condition, run timing, spawn timing, and secondary sexual characteristics in hatchery and wild‐born adults. Second, our ability to evaluate the cause of the shift in age at maturity was hampered by the lack of information about the age distribution of hatchery versus wild fry and smolts in Auke Lake and because we based our inferences about freshwater age from a small subsample of individuals for which scale ages were available and were consistent with ages determined from parentage. Samples meeting those criteria represented about 75% of the samples with readable scales, reflecting uncertainty regarding scale age, parentage assignment, or some combination of the two, as well as the greater stringency for accepting parent assignments for the phenotypic analyses. Using scales to age sockeye salmon is error‐prone, particularly for the freshwater annuli. The last time scale ages were validated in Auke Lake sockeye salmon was 1987 (J. Russell, NOAA, Unpublished data) and given the considerable environmental change Auke Lake has experienced since then (Kovach et al., 2014), an updated scale validation is warranted.

We expect that with the nearly complete genetic sampling conducted in this study, most true parents were sampled and assigned over a close relative, but undoubtedly the parentage assignment was not 100% accurate. Our marker panel had high power to distinguish parent–offspring pairs from unrelated dyads, but the simulations indicated less power for discerning parent–offspring from avuncular relationships (Figure S2). The “aunt and uncle effect” can complicate parentage assignment in salmonids (Olsen et al., 2001) but it is more of a problem when sampling is sparse. We suspect that two of the STR loci harbored null alleles, which would also reduce the accuracy of parentage assignment, but this effect was ameliorated to some extent by using probabilistic parentage assignment, which allows for some mismatches in parent and offspring genotypes, as opposed to strictly exclusion‐based assignment. While parentage errors would add some uncertainty to productivity estimates, we had no reason to think that errors were distributed disproportionately between wild and hatchery offspring, so should have had minimal impact on the comparisons made.

## 5 CONCLUSIONS

Using near‐census adult sampling, genotyping, and parentage assignment, we documented very high productivity (returning adults/spawner) resulting from captive spawning of sockeye salmon from Auke Lake, Alaska over three experimental brood years. The productivity of female hatchery spawners ranged from 6 to ≈50 times that of wild females, indicating that supplementation of sockeye salmon can effectively increase the number of returning adults. The success of this type of supplementation depends on the characteristics of the sockeye salmon system, though. Supplementation by captive spawning and subsequent release into the rearing lake is expected to be counterproductive in systems that are limited by lake rearing rather than spawning and incubation habitat (Hyatt et al., 2005).

In Auke Lake sockeye salmon, supplementation produced no detectable effect on run timing and affected size at age in only one of the three brood years, but it did cause a loss of age diversity and a shift toward younger fish within a single generation that was consistent over all three brood years. This finding raises concern because a typical goal of such programs is to minimize the effects on the recipient wild population, and because reductions in average age could exacerbate negative effects of supplementation on effective population size. Although we could not identify the cause of the shift in age distribution, scale‐based ages indicated that hatchery fish tended to spend fewer years in freshwater than did wild fish. Future studies investigating the role of captive spawning and rearing on early growth and propensity to emigrate would allow hatchery managers to better mitigate for potential phenotypic effects of supplementation.

Ultimately, managers must weigh concerns over potentially negative effects of supplementation, such as phenotypic shifts, effective population size, and fitness, against the demographic gains. For example, natural recruitment in Auke Lake sockeye salmon was below replacement in two of the three brood years of our study. Based on the values presented in Table 2, the females removed for broodstock in 2012 and 2013 would be expected to contribute 20 and 23 returning adults, respectively, had they spawned in the wild; instead, they contributed substantially to the adult population size (producing 701 and 286 returning adults, respectively). This study demonstrates that when captive spawning can mitigate a critical habitat limitation without contributing to density dependence, it can be an effective intervention to prevent short‐term population decline. Parentage assignment is an effective way to assess both demographic efficacy and evolutionary consequences when strategizing the optimal use of hatchery supplementation.

## FUNDING INFORMATION

Funding for this study was provided by the Pacific Salmon Commission Northern Fund.

## CONFLICT OF INTEREST STATEMENT

The authors declare no conflicts of interest.

## Supporting information


Appendix S1

## Data Availability

The code that underlies the findings of this research is available at https://github.com/mvmcphee/Auke_sockeye_F1_RP. The data are publicly available via Dryad (doi:10.5061/dryad.vhhmgqp1p).

## Endnotes

^1^

https://www.psc.org/publications/technical‐reports/technical‐committee‐reports/transboundary/.

^2^
Juneau Watershed Partnership, 2009. Auke Lake watershed assessment. https://doi.org/10.3996/112014‐JFWM‐083.S15.
