# Supplementary material for: Hatchery supplementation provides a demographic boost but alters age composition of sockeye salmon in Auke Lake, Southeast Alaska
Source: Evol Appl. 2024 Feb 7;17(2):e13640. doi: 10.1111/eva.13640 (PMC10848869; doi:10.1111/eva.13640)
Supplement: Supplementary file 1 — Appendix S1 [file EVA-17-e13640-s001.docx]

**Hatchery supplementation provides a demographic boost but alters age composition of sockeye salmon in Auke Lake, Southeast Alaska**

Supplementary Materials

**Supplementary Table S1.** Name, type (short tandem repeat, STR; single nucleotide polymorphism, SNP), and original source of the loci we genotyped for parentage analysis.

| **Locus** | **Locus type** | **Source** |
| --- | --- | --- |
| *Oki10* | STR | Smith et al. 1998 |
| *One109* | STR | Olsen et al. 2000 |
| *Oki1a* | STR | Smith et al. 1998 |
| *Oki1b* | STR | Smith et al. 1998 |
| *One8* | STR | Scribner et al. 1996 |
| *Oki100* | STR | Beacham et al. 2008 |
| *One102* | STR | Olsen et al. 2000 |
| *One114* | STR | Olsen et al. 2000 |
| *Ssa419* | STR | Cairney et al. 2000 |
| *One_ACBP-79* | SNP | Elfstrom et al. 2006 |
| *One_agt-132* | SNP | Storer et al. 2012 |
| *One_apoe-83* | SNP | Storer et al. 2012 |
| *One_cetn1-167* | SNP | Storer et al. 2012 |
| *One_cin-177* | SNP | Campbell & Narum 2011 |
| *One_dds-529* | SNP | Campbell & Narum 2011 |
| *One_DDX5-86* | SNP | Storer et al. 2012 |
| *One_E2* | SNP | Smith et al. 2005 |
| *One_gdh-212* | SNP | Campbell & Narum 2011 |
| *One_GHII-2461* | SNP | Elfstrom et al. 2006 |
| *One_GPH-414* | SNP | Elfstrom et al. 2006 |
| *One_hcs71-220* | SNP | Elfstrom et al. 2006 |
| *One_HGFA* | SNP | Smith et al. 2005 |
| *One_HpaI-99* | SNP | Elfstrom et al. 2006 |
| *One_Hsp47* | SNP | Miller & Beacham (2007) |
| *One_IL8r-362* | SNP | Habicht et al. 2010 |
| *One_KCT1-453* | SNP | Storer et al. 2012 |
| *One_LEI-87* | SNP | Elfstrom et al. 2006 |
| *One_MHC2_251* | SNP | Elfstrom et al. 2006 |
| *One_Mkpro-129* | SNP | Campbell & Narum 2011 |
| *One_ODC1-196* | SNP | Storer et al. 2012 |
| *One_Ots208-234* | SNP | Campbell & Narum 2011 |
| *One_Ots213-181* | SNP | Elfstrom et al. 2006 |
| *One_PIP* | SNP | Miller & Beacham (2007) |
| *One_Prl2* | SNP | Elfstrom et al. 2006 |
| *One_psme2-354* | SNP | Storer et al. 2012 |
| *One_STC-410* | SNP | Elfstrom et al. 2006 |
| *One_STR07* | SNP | Elfstrom et al. 2006 |
| *One_SUMO1-6* | SNP | Campbell & Narum 2011 |
| *One_Tf_ex10-750* | SNP | Elfstrom et al. 2006 |
| **Locus** | **Locus type** | **Source** |
| *One_U1004-183* | SNP | Storer et al. 2012 |
| *One_U1012-68* | SNP | Storer et al. 2012 |
| *One_U1016-115* | SNP | Storer et al. 2012 |
| *One_U1024-197* | SNP | Storer et al. 2012 |
| *One_U1201-492* | SNP | Storer et al. 2012 |
| *One_U1202-1052* | SNP | Storer et al. 2012 |
| *One_U1206-108* | SNP | Storer et al. 2012 |
| *One_U1208-67* | SNP | Storer et al. 2012 |
| *One_U1210-173* | SNP | Storer et al. 2012 |
| *One_U1212-106* | SNP | Storer et al. 2012 |
| *One_U1215-82* | SNP | Storer et al. 2012 |
| *One_U1216-230* | SNP | Storer et al. 2012 |
| *One_U301-92* | SNP | Elfstrom et al. 2006 |
| *One_U401-224* | SNP | Habicht et al. 2010 |
| *One_U504-141* | SNP | Habicht et al. 2010 |

Sources:

Beacham, T., McIntosh, B., MacConnachie, C., Miller, K., Withler, R., and Varnavskaya, N. 2006. Pacific rim population structure of sockeye salmon as determined from microsatellite analysis. Trans. Am. Fish. Soc. **135**(1): 174–187.

Cairney, M., Taggart, J. B., and Hoyheim, B. 2000. Characterization of microsatellite and minisatellite loci in Atlantic salmon (*Salmo salar* L.) and cross-species amplification in other salmonids. Mol. Ecol. **9**: 2175-2178.

Olsen, J.B., Wilson, S.L., Kretschmer, E.J., Jones, K.C., and Seeb, J.E. 2000. Characterization of 14 tetranucleotide microsatellite loci derived from sockeye salmon. Mol. Ecol. **9**:2185-2187.

Smith, C.T., Koop, B.F., and Nelson, R.J. 1998. Isolation and characterization of coho salmon (*Oncorhynchus kisutch*) microsatellites and their use in other salmonids. Mol. Ecol. **7**:1614-1616.

Scribner, K.T., Gust, J.R., and Fields, R.L. 1996. Isolation and characterization of novel salmon microsatellite loci: Cross-species amplification and population genetic applications. Can. J. of Fish. Aquat. Sci. **53**:833-841.

**Supplementary Table S2.** Number of individual tissue samples collected by brood year and spawning type and the number (percent) of individuals that were successfully genotyped at all 54 loci and at least 40 loci.

|  |  |  | Successfully genotyped (%) | |
| --- | --- | --- | --- | --- |
| Brood year | Parent type | N collected | All 54 loci | ≥ 40 loci |
| 2011 | hatchery | 41 | 87.8 | 97.6 |
|  | wild | 2,382 | 88.0 | 98.7 |
| 2012 | hatchery | 32 | 75.0 | 96.9 |
|  | wild | 1,524 | 73.2 | 93.6 |
| 2013 | hatchery | 42 | 57.1 | 97.6 |
|  | wild | 2,014 | 62.1 | 98.6 |

**Supplementary Table S3.** Overall productivity, expressed as the total number of offspring divided by total number of potential parents (N), by parental type (hatchery or wild) brood year, and LOD threshold for accepting parent assignments. Also shown is relative productivity (hatchery productivity/wild productivity) by LOD threshold.

|  |  | Threshold for parent-offspring assignment | | |  |
| --- | --- | --- | --- | --- | --- |
| Brood year | Type | None | LOD ≥ 4.5 | LOD ≥ 9 | N |
| 2011 | hatchery | 14.35 | 14.35 | 13.85 | 40 |
|  | wild | 1.63 | 1.62 | 1.55 | 2,351 |
|  | RP | 8.80 | 8.86 | 8.93 |  |
| 2012 | hatchery | 23.32 | 23.29 | 22.97 | 31 |
|  | wild | 0.41 | 0.40 | 0.37 | 1,427 |
|  | RP | 56.88 | 58.23 | 62.08 |  |
| 2013 | hatchery | 7.12 | 7.10 | 6.80 | 41 |
|  | wild | 0.44 | 0.42 | 0.37 | 1,985 |
|  | RP | 16.18 | 16.90 | 18.38 |  |

**Supplementary Table S4.** Results of linear models for each brood year to test whether size at age differs by origin (hatchery or wild) after accounting for sex and age.

|  | 2011 | | 2012 | | 2013 | |
| --- | --- | --- | --- | --- | --- | --- |
| Parameter | Estimate (SE) | *p* | Estimate (SE) | *p* | Estimate (SE) | *p* |
| Intercept | 467.3 (8.1) | 2x10^-16^ | 472.8 (10.3) | 2x10^-16^ | 495.4 (13.8) | 2x10^-16^ |
| Origin (wild) | 4.6 (4.7) | 0.33 | 0.5 (5.6) | 0.93 | -14.3 (5.2) | 0.007 |
| Sex (male) | 18.2 (2.8) | 4.6x10^-10^ | 14.3 (5.0) | 0.005 | 27.3 (4.4) | 8.2x10^-9^ |
| Scale age (1.3) | 56.6 (7.5) | 6.4x10^-13^ | 53.8 (10.7) | 2.3x10^-6^ | 36.0 (14.0) | 0.01 |
| Scale age (2.2) | 14.4 (7.7) | 0.06 | 16.8 (11.1) | 0.13 | 19.0 (13.7) | 0.17 |
| Scale age (2.3) | 45.2 (7.3) | 2.9x10^-9^ | 73.1 (12.0) | 2.7x10^-8^ | 32.3 (14.0) | 0.03 |

**Supplementary Figure S1.** Phenotypic comparisons of hatchery (pink) and wild (blue) spawners by brood year: a) body length; b) date of capture at the Auke Creek weir. The crosses on panel b indicate the days when individuals used as hatchery broodstock were captured.

**Supplementary Figure S2**. Results of simulations conducted in CKMRsim assuming a genotyping error rate of 0.005, showing a) the distribution of LOD scores for parent-offspring (PO) and unrelated (U) individuals for the full marker set; b) the product of false negative (FNR) and false positive (FPR) error rates as a function of LOD threshold for the full marker set; c) the distribution of LOD scores including avuncular (AN) relationships for the full marker set; and d) the distribution of LOD scores for parent-offspring and unrelated individuals using only SNPs.

**Supplementary Figure S3.** Distribution of LOD scores for all putative (i.e., LOD > 0) parent-offspring assignments by brood year and parental type. Median values are depicted by the white circle.

**
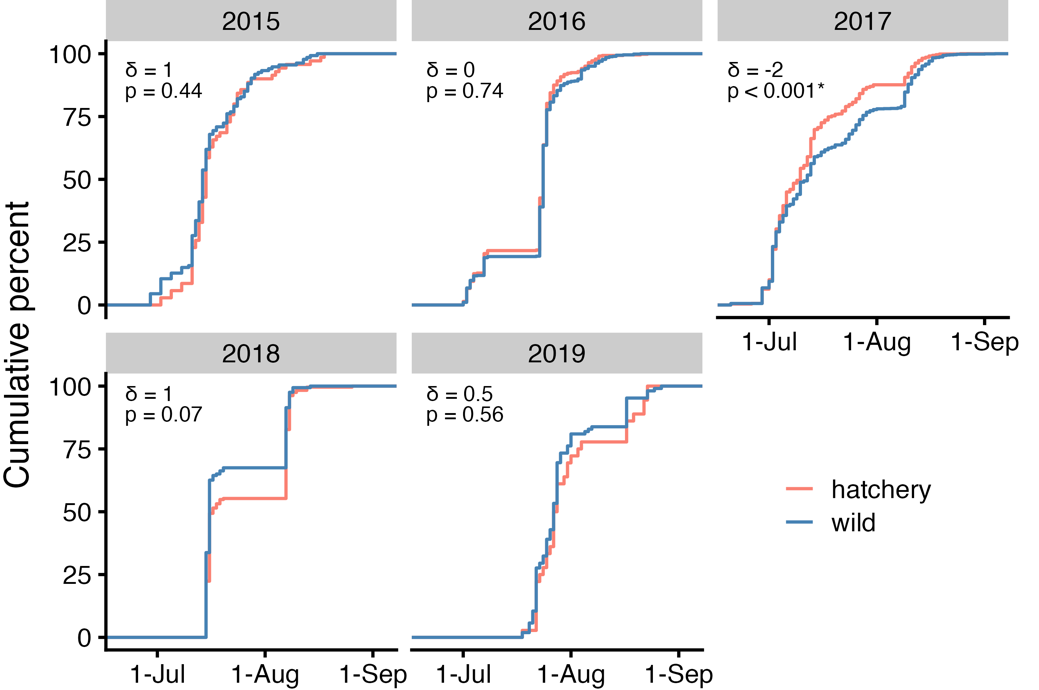
**

**Supplementary Figure S4.** Date of capture at the Auke Creek weir of returning adult offspring of hatchery parents and of wild parents that were sampled at weir on the same dates that hatchery parents were captured, by offspring return year (2015-2019). Also shown are δ (the difference between the median dates of hatchery versus wild-origin offspring, where a negative value means the median date of hatchery-origin returns is earlier) and *p* (the probability of a difference between median date of return (two-sided Kolmogorov-Smirnov test).

**
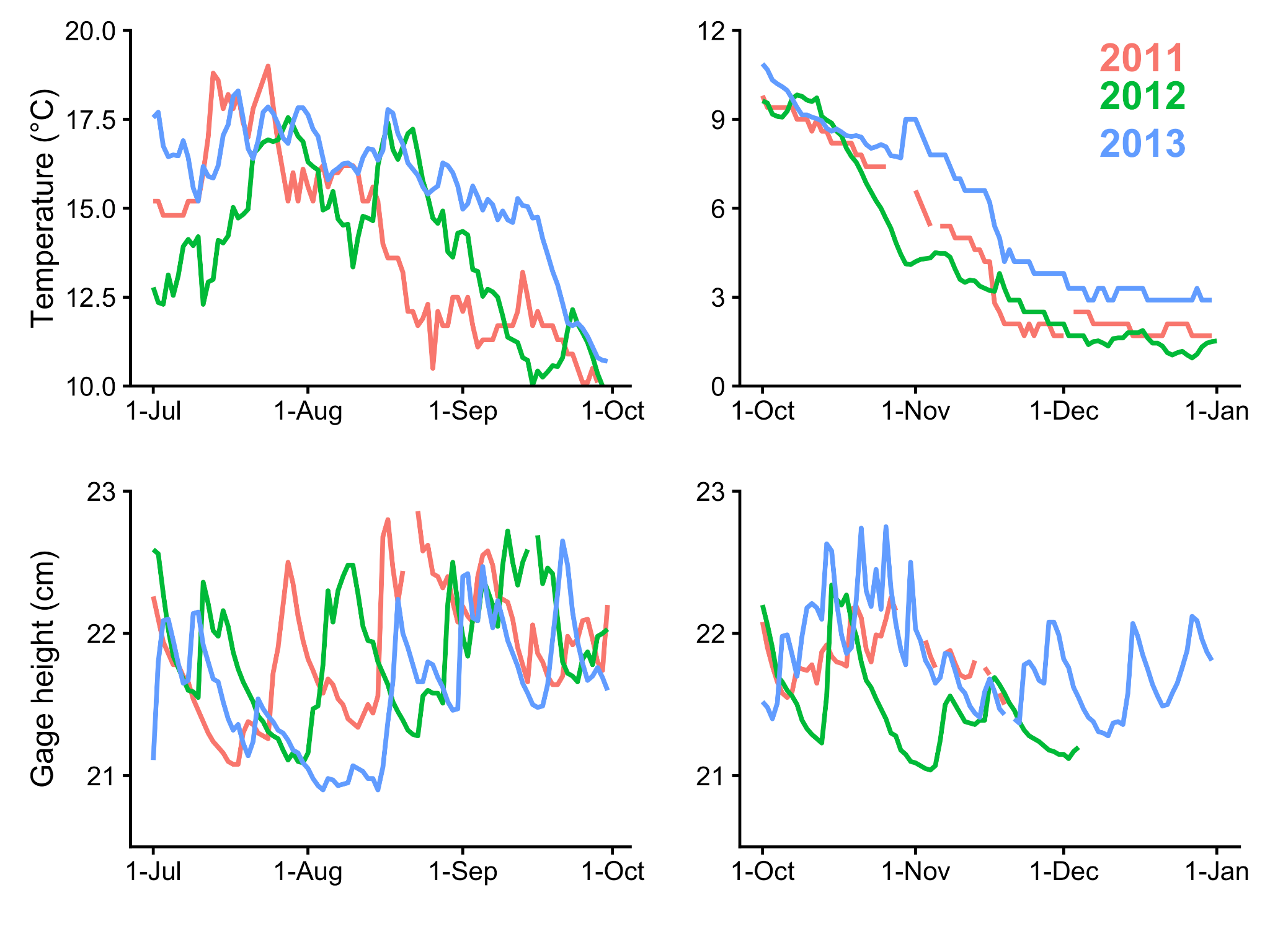
**

**Supplementary Figure S5**. Daily measurements of Auke Creek temperature and gage height for the migration and spawning periods (1 July – 30 September, left-hand panes) and incubation periods (1 October – 31 December, right-hand panes) by year. Note difference in temperature scale between migration/spawning and incubation periods.
